# A case of small bowel obstruction caused by konjac

**DOI:** 10.1093/jscr/rjag277

**Published:** 2026-04-17

**Authors:** Yoshitaka Ooya, Masahito Kaji

**Affiliations:** Departments of Emergency and Acute Medicine, Saitama Medical University International Medical Center, 1397-1 Yamane, Hidaka City, Saitama, Japan; Departments of Emergency and Acute Medicine, Saitama Medical University International Medical Center, 1397-1 Yamane, Hidaka City, Saitama, Japan

**Keywords:** small bowel obstruction, food-induced bowel obstruction, konjac, phytobezoar, glucomannan

## Abstract

While postoperative adhesions, hernias, and tumors are common causes of small bowel obstruction (SBO), food-induced obstruction is rare. Konjac (*Amorphophallus konjac*)–related SBO is exceptionally uncommon, with only a few cases documented in the English literature. We present the case of a man in his 50s who developed abdominal pain during breakfast. After conservative management at a local clinic failed, he was referred to our hospital. Abdominal computed tomography indicated SBO of unknown etiology. Emergency laparotomy revealed four connected pieces of konjac obstructing the small intestine. The patient recovered uneventfully and was discharged on postoperative day 8. This case represents a rare instance of konjac-induced SBO. Given the rising global consumption of konjac products—especially in Western countries—clinicians should consider konjac ingestion as a potential cause of food-induced bowel obstruction.

## Introduction

Small bowel obstruction (SBO) is a frequent surgical emergency, typically caused by postoperative adhesions, tumors, or hernias [1, 2]. In rare cases, ingested food material can act as an obstructive agent, leading to what is termed food-induced bowel obstruction (FIBO) [3]. Foods implicated in FIBO include dried persimmons, shiitake mushrooms, dried apricots, and dried apples—all high in indigestible fiber [4–6].

Konjac (*Amorphophallus konjac*) is rich in water-soluble dietary fiber, particularly glucomannan. Its dense and elastic consistency makes it prone to forming intraluminal masses when inadequately chewed. Choking incidents involving konjac jelly have prompted safety warnings from health authorities [7]. However, intestinal obstruction due to konjac remains extremely rare.

Traditionally consumed in East Asia, konjac has gained popularity worldwide as a low-calorie, gluten-free, plant-based food. Shirataki noodles, konjac jelly, and konjac-based supplements are now widely available in global markets [8].

Furthermore, konjac is increasingly used in vegetarian and vegan diets as a meat substitute. Products such as ‘konjac steak’ or ‘konjac-based meat analogues’ have a thick, resilient texture that can pose a risk for intestinal blockage if not adequately chewed. Elderly individuals, denture wearers, and patients with cognitive impairment may be especially vulnerable. Chewing dysfunction is a well-recognized risk factor for FIBO [6, 8].

Here, we describe a rare case of mechanical SBO caused by konjac ingestion, highlighting the clinical significance of this underrecognized etiology in the context of expanding global consumption.

## Case report

**Figure 1:**
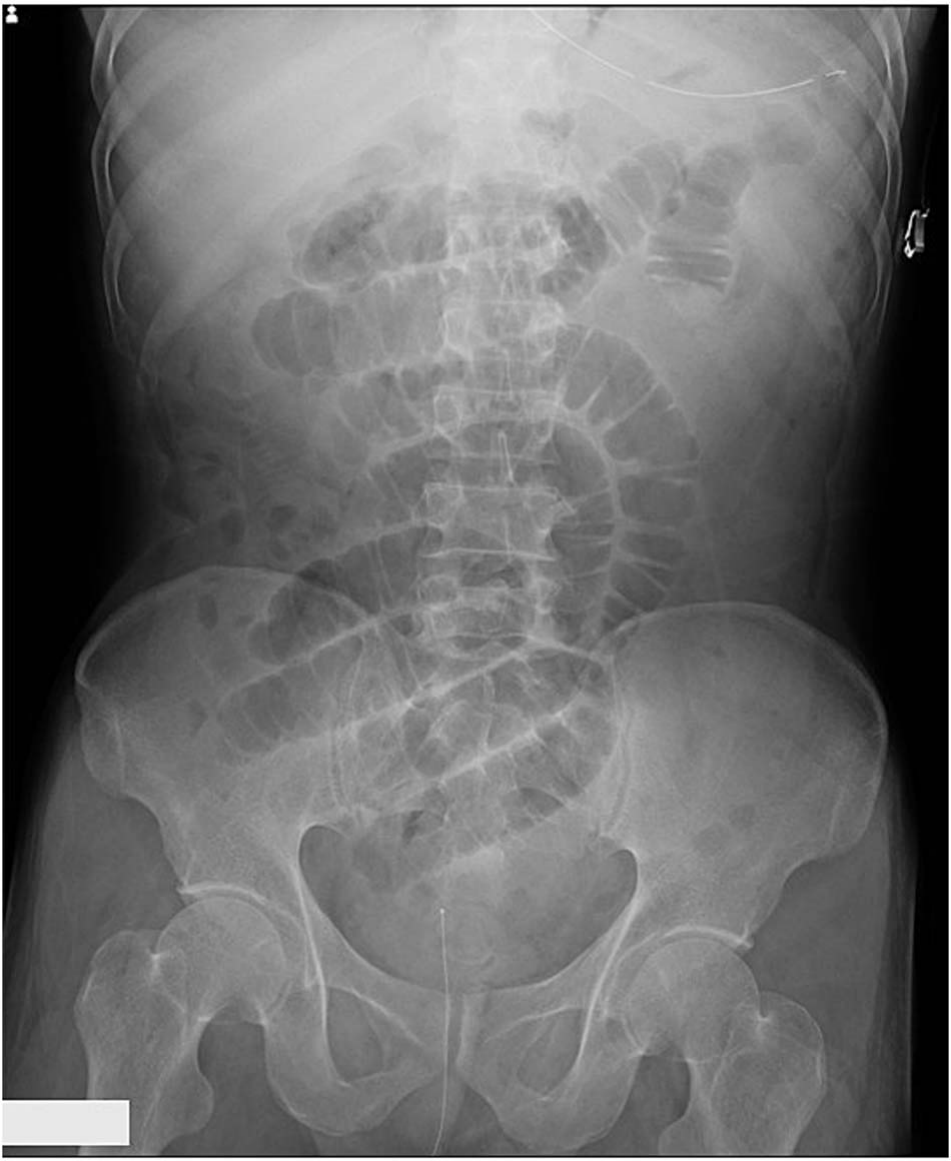
Supine abdominal X-ray showing dilated small bowel loops.

**Figure 2:**
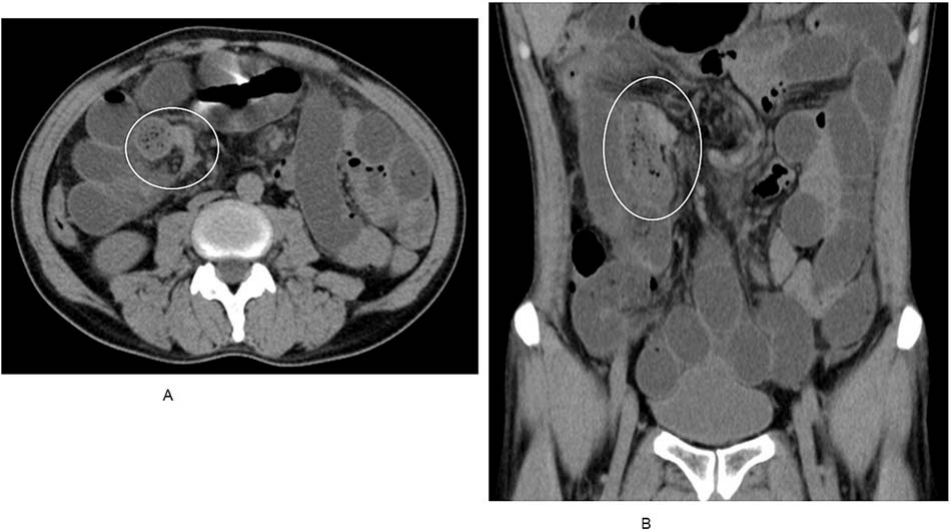
(A) Axial CT scan revealing a small bowel feces sign in the right upper quadrant. (B) Coronal CT view demonstrating collapsed distal bowel loops beyond the obstruction.

**Figure 3:**
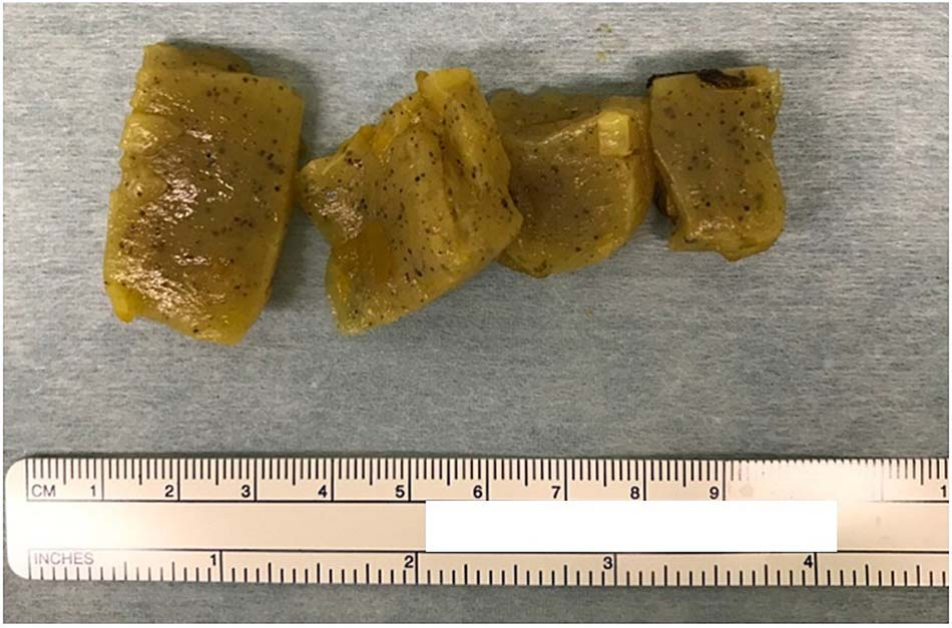
Four connected konjac fragments (each ~2 cm) removed via enterotomy.

A man in his 50s presented with abdominal pain after developing periumbilical pain at ~5:30 a.m. while eating breakfast. He initially visited a local clinic, where he was treated with digestive agents and antiemetics; however, his symptoms persisted, and he was transferred to our emergency department later that evening. On arrival, he was alert and hemodynamically stable, with a blood pressure of 150/80 mmHg, heart rate of 90 beats/min, and oxygen saturation of 98% on room air. Abdominal examination revealed distension and periumbilical tenderness with hyperactive bowel sounds, without signs of peritonitis. A supine abdominal radiograph showed dilated small bowel loops (Fig. 1). Although air-fluid levels were not clearly visualized, abdominal computed tomography demonstrated a small bowel feces sign in the right upper quadrant and collapse of the distal bowel loops (Fig. 2a and b). No definite obstructing lesion was identified, and small bowel obstruction of unknown etiology was diagnosed. Emergency laparotomy was performed. Intraoperatively, a firm intraluminal mass was palpated ~70 cm proximal to the ileocecal valve. Enterotomy revealed four connected fragments of konjac, each measuring ~2 cm (Fig. 3). The enterotomy was closed, the peritoneal cavity was irrigated, and the abdomen was closed in layers. Estimated blood loss was 10 mL, and the operative time was 36 min. The postoperative course was uneventful, and the patient was discharged on postoperative day 8.

## Discussion

FIBO accounts for an estimated 1.9%–4% of all SBO cases [3]. Common causative foods are rich in fiber, expand in the intestine, and resist digestion, such as dried fruits and mushrooms [4–6].

Konjac, composed primarily of glucomannan, is poorly digested and highly elastic. When inadequately chewed, it may pass into the gastrointestinal tract intact and obstruct the bowel lumen [6]. In our case, the patient had consumed multiple konjac pieces that likely remained undigested and formed a mass sufficient to cause SBO. The obstructive material was not identified preoperatively, emphasizing the diagnostic challenge in such cases.

With the international popularity of konjac increasing, particularly as a health food, the risk of FIBO associated with konjac should not be overlooked. In addition to its use in traditional Asian cuisine, newer forms such as konjac steak and meat substitutes are becoming more accessible globally [8].

Risk factors for FIBO include denture use, poor dentition, prior gastric surgery, advanced age, and rapid eating habits [3, 9]. Although our patient had no significant medical history, chewing insufficiency was considered a contributing factor.

This case underscores the importance of dietary counseling and the need for clinicians to recognize unconventional causes of SBO in an era of increasingly diverse food practices.

## Conclusion

Although rare, small bowel obstruction caused by konjac ingestion should be included in the differential diagnosis of food-related intestinal blockages. As konjac-based products continue to proliferate in global markets, raising awareness of their potential risks is essential for both healthcare providers and the public.
